# Cytokine profiles and CD4 counts in HIV-positive individuals with cysticercosis: implications for sex-specific immune responses in co-endemic regions of Tanzania

**DOI:** 10.3389/fimmu.2025.1521295

**Published:** 2025-02-19

**Authors:** Yakobo Leonard Lema, Ulrich Fabien Prodjinotho, Charles Makasi, Mary-Winnie A. Nanyaro, Andrew Martin Kilale, Sayoki Godfrey Mfinanga, Veronika Schmidt, Hélène Carabin, Andrea Sylvia Winkler, Eligius F. Lyamuya, Bernard James Ngowi, Clarissa Prazeres da Costa

**Affiliations:** ^1^ Muhimbili Medical Research Center, National Institute for Medical Research (NIMR), Dar es Salaam, Tanzania; ^2^ Institute for Medical Microbiology, Immunology, and Hygiene, School of Medicine and Health, Technical University of Munich (TUM), Munich, Germany; ^3^ Center for Global Health, School of Medicine and Health, Technical University of Munich, Munich, Germany; ^4^ Institute of Public Health, Kilimanjaro Christian Medical University College, Moshi, Tanzania; ^5^ Department of Public Health, Kampala International University, Dar Es Salaam, Tanzania; ^6^ School of Public Health, Muhimbili University of Health and Allied Sciences (MUHAS), Dar Es Salaam, Tanzania; ^7^ Faculty of Veterinary Medicine, University of Montreal, Saint-Hyacinthe, QC, Canada; ^8^ School of Public Health, University of Montreal, Montreal, QC, Canada; ^9^ Research Group on Epidemiology of Zoonoses and Public Health (GREZOSP), Saint-Hyacinthe, QC, Canada; ^10^ Department of Epidemiology, Public Health Research Center of the Centre intégré universitaire de santé et de services sociaux (CIUSSS) of Center-Sud-de-l’le-de-Montréal (CReSP), Montreal, QC, Canada; ^11^ Department of Community Medicine and Global Health, Institute of Health and Society, University of Oslo, Oslo, Norway; ^12^ Department of Global Health and Social Medicine, Harvard Medical School, Boston, MA, United States; ^13^ Department of Microbiology and Immunology, Muhimbili University of Health and Allied Sciences (MUHAS), Dar Es Salaam, Tanzania; ^14^ Mbeya College of Health and Allied Sciences, University of Dar Es Salaam, Mbeya, Tanzania; ^15^ Department of Infectious Diseases, German Center for Infection Research (DZIF), Partner Site Munich, Munich, Germany

**Keywords:** *Taenia solium* cysticercosis, HIV, cytokine profiles, CD4 count, sex-dependent responses

## Abstract

**Introduction:**

The interplay between HIV and *Taenia solium* cysticercosis in co-endemic regions remains poorly understood, particularly regarding the immune responses but is relevant for an effective treatment strategy. This study aimed to investigate the relationship between peripheral cytokine profiles, CD4^+^ T-cell counts, and cysticercosis infection status in HIV-positive individuals in Tanzania’s southern highlands.

**Methods:**

We conducted a cross-sectional study involving 110 HIV-positive individuals. Cysticercosis was diagnosed using antibody and antigen tests, with neurocysticercosis confirmed by CT imaging. CD4 counts and serum cytokine levels (pro- and anti-inflammatory) were analyzed using multivariate regression and MANOVA, including sex-stratified analyses.

**Results:**

Among participants, 20.9% tested positive for cysticercosis antibodies, 43.6% for antigens and 20.2% presented with brain cysts, with 10.6% showing active neurocysticercosis. Cysticercosis-positive individuals showed positive correlations between CD4 counts and pro-inflammatory cytokines (e.g., TNF-α, IL-1β), contrasting with negative correlations in cysticercosis-negative individuals. Sex-stratified analysis showed stronger regulatory cytokine responses in males compared to females, particularly involving higher levels of IL-10 and IL-4 indicating sex-specific immune modulation in co-infected individuals. However, overall cytokine profiles were not significantly influenced by CD4 categories or cysticercosis status.

**Conclusion:**

These results contribute to our understanding of immunological interactions in HIV-cysticercosis co-infection and underscore the need for further research with larger sample sizes to elucidate the clinical implications of these findings. Such studies could inform the development of more effective, sex-personalized treatment strategies for HIV patients in cysticercosis-endemic regions.

## 1 Introduction

*Taenia solium*, a zoonotic parasite, significantly impacts human and animal health in many low-income countries, leading to substantial economic losses and healthcare burden (1, 2). Neurocysticercosis (NCC), caused by presence of the larval stage of *T. solium* in the central nervous system (CNS), is recognized as a leading cause of epilepsy and is associated with significant morbidity and mortality in the endemic areas especially in sub-Sahara Africa (SSA) (2, 3). According to the WHO Foodborne Disease Burden Epidemiology Reference Group, NCC is the third leading cause of death from food-borne diseases, resulting in an estimated 2.8 million disability-adjusted life-years (DALYs) (4).

In humans, while the adult tapeworm resides in the intestines causing taeniasis, the larval cysticerci can infect various tissues causing cysticercosis (CC), including CNS leading to NCC (5, 6). NCC can present as symptomatic or asymptomatic disease, with up to 50% of cases being asymptomatic, contributing to underdiagnosis and continued transmission of the parasite (7–9). When symptomatic, the most common manifestations include severe progressive headaches and epileptic seizures (acute or chronic), which can be associated with reduced quality of life, and social stigma (10–12). Seroprevalence studies have shown significant regional variation in *T. solium* infection rates. In SSA, community surveys using antigen ELISA reported prevalences ranging from 5.8% to 22% (9, 13, 14). In northern Tanzania, a hospital-based cross-sectional study reported definitive and probable NCC in 2.4% and 11.3% of people with epilepsy, respectively (6, 15).

Many *T. solium* endemic areas in SSA are also disproportionately burdened by the human immunodeficiency virus (HIV). Approximately 25 million people living with HIV/AIDS reside in SSA which is also home to an estimated 3 million people suffering from NCC-related epilepsy (2, 6, 16). Thus, the interplay between HIV and parasitic infections is an important area of research, with studies showing that some parasitic infections can enhance susceptibility to HIV and potentially accelerate disease progression (17).

While interactions between HIV and certain soil-transmitted parasitic infections have been well-documented, the main findings indicate that helminths are potent immunomodulators, suggesting that HIV patients might benefit from anthelminthic treatment (18, 19). However, the relationship between HIV and CC, particularly NCC, remains less understood (20–23).

Studies from Mexico have demonstrated significant immunological alterations in patients with NCC. Fleury et al. found that viable cysticerci release immunomodulatory molecules that induce a shift towards a Th2-type immune response, characterized by increased levels of anti-inflammatory cytokines such as interleukin-4 (IL-4) and interleukin-10 (IL-10) (24). This shift can suppress the pro-inflammatory Th1 response, leading to decreased production of cytokines like interferon-gamma (IFN-γ) and interleukin-2 (IL-2), which are crucial for cell-mediated immunity. This suppression may jeopardize anti-bacterial and anti-viral immune responses. Restrepo et al. observed that NCC patients exhibit altered cytokine profiles with elevated IL-5 and IL-6 levels, alongside reduced IFN-γ production, suggesting a compromised immune response (25). These immunological changes could contribute to the persistence of the parasite and exacerbate immunosuppression.

Research findings on the interaction between HIV and NCC have been varied and inconclusive. Some evidence suggests that HIV-associated immunosuppression-particularly in individuals with CD4^+^ T-cell counts below 200 cells/mm³, may influence *T. solium* infection, leading to more asymptomatic cases due to a reduced inflammatory response (26–28). However, the relationship between low CD4+ counts and an increased risk of NCC infection remains uncertain, with no clear consensus as to whether immunosuppression increases susceptibility or merely alters symptomatology. Noormahomed et al. (2014) found no significant correlation between CC antibodies and CD4^+^ T-cell counts in HIV patients (29). Additionally, a study by Schmidt et al. (2016) did not observe a higher prevalence of CC in HIV-positive individuals compared to HIV-negative controls (6).

Regarding NCC, multiple studies have demonstrated that HIV infection is not associated with a higher prevalence of NCC, and in some cases, the prevalence may even be lower compared to HIV-negative populations (6, 29–33). These findings highlight the need for further research into how HIV-associated immunosuppression could alter the course of *T. solium* infection, and thereby CC disease prevalence, symptoms, and treatment outcomes (34). Specifically, HIV-positive patients with higher CD4+ T-cell counts may be more likely to exhibit symptomatic NCC when they acquire the infection due to a more robust immune response leading to inflammation, whereas immunocompromised patients might present asymptomatically or with atypical forms of the disease (35, 36). To investigate the peripheral immune responses and clinical presentation in relation to CD4 T cell counts in HIV and CC/NCC co-infected patients, we recently conducted a study in Southern Tanzania and could show that HIV infection itself significantly modulates levels of key cytokines such as TNF-α, IL-8, and IFN-γ (20). However, HIV did not alter cytokine levels induced by CC or NCC. These immunological findings highlight the complex interplay between HIV and NCC but leave unclear how CC-induced cytokine profiles might influence CD4^+^ T-cell counts in HIV-infected individuals.

The present study addresses this gap by investigating the influence of cytokine profiles modulated by CC infection on CD4^+^ T-cell counts in HIV-positive patients in Tanzania’s southern highlands.

## 2 Materials and methods

### 2.1 Study design and participants

We conducted a nested cross-sectional immunological study within a larger paired cross-sectional investigation in the Southern Highlands of Tanzania between June 2018 and March 2021. The study focused on Chunya district (Mbeya region) and Iringa rural district (Iringa region), areas characterized by high HIV prevalence (9.0% and 9.1%, respectively) and significant porcine CC prevalence (7.6% and 8.4%, respectively) (37).

The parent study recruited 1,291 people living with HIV/AIDS (PLWH/A) on antiretroviral therapy (ART) aged 14 years and above from HIV clinics in the study regions. Exclusion criteria included opportunistic infections, neurological symptoms predating HIV diagnosis, and anthelminthic treatment within the previous 12 months. All participants underwent clinical evaluation, neurological examination, and serological testing for *T. solium* CC.

For our nested investigation, we included 110 HIV-positive participants from the parent study who had complete data on CC serological status, CD4 counts, cytokine profiles, and CT scan examinations, allowing for a comprehensive analysis of the interplay between HIV infection, (neuro-)cysticercosis, and immune responses.

### 2.2 Ethics statement

This cross-sectional study was conducted in accordance with the Declaration of Helsinki. Ethical approval was obtained from the Directorate of Research and Publications at Muhimbili University of Health & Allied Sciences (MUHAS), Dar es Salaam (Ref.No.DA.282/298/01.C/), and the National Institute for Medical Research (NIMR) in Tanzania (Ref. NIMR/HQ/R.8a/Vol. IX/2529) as part of the broader Cystinet Africa project. The Technical University of Munich, School of Medicine and Health, Ethics Committee also provided clearance (Ref: 537/18 and 215/18S).

All participants provided written informed consent prior to enrollment. Participants were informed about the study procedures, potential risks, and benefits, including those associated with diagnostic tests and CT imaging where applicable. The study protocol ensured appropriate care for PLWHA participants throughout the research process. Participants’ privacy and confidentiality were maintained through data anonymization during collection and analysis. The study design prioritized the well-being and rights of people living with HIV, adhering to principles of respect for autonomy, beneficence, and justice. No children or pregnant women were included in this study.

### 2.3 Sample collection and handling

Venous blood samples (15 mL) were collected from each participant using standard aseptic technique. Blood was drawn into two types of Vacutainer tubes (BD Biosciences, Franklin Lakes, NJ, USA): a 5 mL Serum Separator Tube (SST) and a 10 mL EDTA tube. For serum collection, SST tubes were inverted 5 times after collection and allowed to clot at room temperature for 30 minutes. Samples were then centrifuged at 1500 x g for 10 minutes at 4°C. The resulting serum was aliquoted into separate vials: 50 µL each for CC antigen testing, antibody testing, and cytokine analysis, with the remaining serum stored in 0.5 mL aliquots. EDTA tubes were inverted 8-10 times immediately after collection to ensure proper mixing with the anticoagulant. These samples were used for CD4 count analysis using the BD FACSPresto™ System (BD Biosciences, San Jose, CA, USA) within 6 hours of collection, and HIV-1 viral load quantification using the Xpert HIV-1 Viral Load Assay on the GeneXpert platform (Cepheid, Sunnyvale, CA, USA) (38).

All aliquots were stored at -80°C within 2 hours of processing. Samples were transported on dry ice to the respective laboratories for analysis. Freeze-thaw cycles were minimized, with samples thawed only once for each analysis.

### 2.4 Diagnosis of NCC and CC

(Neuro-) Cysticercosis diagnosis was based on serological testing and neuroimaging. We employed two serological tests: the CYSTICERCOSIS Western Blot (WB) IgG test (LDBio Diagnostics SARL, Lyon, France) for antibody detection and the CC Ag ELISA (apDia bvba, Turnhout, Belgium) for circulating antigen detection.

The WB IgG test utilizes antigens from *T. solium* cysticerci of porcine origin, separated by electrophoresis and bound to nitrocellulose strips. Serum samples (1:50 dilution) were incubated with individual strips for 90 minutes at 20-26°C. After washing, alkaline phosphatase-anti human IgG conjugate was added and incubated for 60 minutes. Following additional wash steps, strips were developed with NBT/BCIP substrate for 60 minutes. The reaction was then stopped, and strips were dried and interpreted. A positive result was indicated by the presence of at least two well-defined bands among five main bands (P6-8, P12, P23-26, P39, and P50-55). This test has a reported sensitivity of 97.5% and specificity of 100% based on manufacturer’s data.

The Ag ELISA detects circulating antigens from viable *Taenia* spp. metacestodes. Serum samples were pre-treated with 5% trichloroacetic acid (1:1 ratio) for 5 minutes at room temperature, then centrifuged at 12,000g for 5 minutes. Supernatants were neutralized with an equal volume of 0.156M carbonate/bicarbonate buffer. Pre-treated samples (100 µL) were added to wells coated with B158C11A10 monoclonal antibodies and incubated for 15 minutes at 37°C. After washing, peroxidase conjugated B60H8A4 monoclonal antibodies were added and incubated for 15 minutes at 37°C. Following washing, chromogen substrate was added and incubated for 15 minutes at room temperature. The reaction was stopped, and absorbance was read at 450 nm within 15 minutes. Results were expressed as an antigen index (Ag Index), with ≥ 1.3 considered positive, ≤ 0.8 negative, and 0.8-1.3 doubtful. This test has a reported sensitivity of 90% and specificity of 98% (39).

Participants with positive serological results underwent cerebral computed tomography (CT) at Mbeya Zonal Referral Hospital. The CT images were evaluated by experienced radiologists and neurologists who were blinded to the serological results. Based on combined serological and imaging findings, participants were classified as NCC, CC or negative for CC.

#### 2.4.1 Case definition of NCC and CC

For this study, we established specific criteria to define CC cases based on the combination of serological and imaging results. NCC was diagnosed in individuals with active, mixed, or calcified cysts in the brain on cerebral CT scan, regardless of serological test results. CC was defined for individuals who had positive results on the antigen ELISA (apDia test), the antibody Western Blot IgG, or both, with a negative CT scan or without CT scan results, this suggests possible CC infection, potentially with viable cysts located outside the central nervous system. Negative cases were those individuals with negative results on all performed tests, including the antigen ELISA, antibody Western Blot IgG, and CT scan when available.

### 2.5 Multiplex cytokine assay

Serum cytokine levels were quantified using a Human Magnetic Luminex Assay (14-Plex) kit (Catalogue # LXSAHM-14, R&D Systems Europe Ltd.) on a Luminex xMAP 100 MAGPIX system (Luminex Corporation, Austin, TX, USA). The multiplex panel included pro-inflammatory and Th1 cytokines (TNF-α, IFN-γ, IL-1β, IL-6, IL-8, IL-12 p40, IL-17A, IL-18), Th2 cytokines (IL-4, IL-5), anti-inflammatory cytokines (IL-10, IL-13), and cell adhesion molecules (VCAM-1/CD106, ICAM-1/CD54).

Serum samples were centrifuged at 16,000 x g for 4 minutes immediately prior to use. Samples were diluted at least 2-fold (75 μL sample + 75 μL Calibrator Diluent RD6-52) as per kit instructions. Briefly, the assay employs color-coded magnetic microspheres pre-coated with analyte-specific antibodies. 50 μL of standard or diluted serum sample was added to each well of a 96-well plate, followed by 50 μL of diluted microparticle cocktail. The plate was incubated for 2 hours at room temperature on a horizontal orbital shaker (800 ± 50 rpm)., This allowed the analytes to bind to the immobilized antibodies. After washing away unbound substances, 50 μL of diluted biotin-antibody cocktail specific to the analytes of interest was added and incubated for 1 hour. Following another wash step, 50 μL of diluted streptavidin-PE was added and incubated for 30 minutes. After a final wash, the microparticles were resuspended in 100 μL of wash buffer for analysis.

The plate was read within 90 minutes using the Luminex MAGPIX analyzer. Instrument settings were as follows: 50 μL sample volume, 50 count/region, and collection of Median Fluorescence Intensity (MFI). Standard curves for each analyte were generated using a five-parameter logistic (5-PL) curve-fit in xPONENT software version 4.3 (Luminex Corporation). Sample concentrations were determined from the standard curve and multiplied by the appropriate dilution factor. To normalize their distribution before statistical analysis, log transformation was applied to the cytokine concentrations.

Quality control measures included the use of kit-provided standards and controls, as well as inter-assay and intra-assay coefficient of variation calculations to assess precision.

### 2.6 CD4 counts and HIV testing

CD4^+^ T-cell counts were measured using the BD FACSPresto™ Near-Patient CD4 Counter System (BD Biosciences, San Jose, CA, USA). Whole blood samples were collected in 10 mL EDTA Vacutainer tubes (BD Biosciences, Franklin Lakes, NJ, USA). Immediately after collection, tubes were inverted 8-10 times to ensure proper mixing with the anticoagulant. CD4 count analysis was performed within 6 hours of collection to ensure sample integrity. For the assay, 30 μL of EDTA-anticoagulated whole blood was added to the inlet port of the BD FACSPresto cartridge containing dried fluorochrome-conjugated antibody reagents. After an 18-minute incubation period at room temperature (20-25°C), the cartridge was inserted into the BD FACSPresto instrument for analysis. The system employs fluorescence microscopy and absorbance spectrophotometry to provide absolute CD4 count (cells/μL), CD4 percentage of lymphocytes, and hemoglobin concentration (g/dL) results within 4 minutes.

Based on the CD4^+^ T-cell counts, participants were categorized into three groups: severe immunosuppression (SIM) (<200 cells/mm³), moderate immunosuppression (MIS) (200–499 cells/mm³), and normal immune status (NIS) (≥500 cells/mm³). This stratification is widely adopted in HIV research and clinical practice and follows the standard guidelines established by both the World Health Organization (WHO) and the Centers for Disease Control and Prevention (CDC), which use similar thresholds to classify the degree of immunodeficiency in HIV-infected individuals. This stratification allowed for the analysis of immune responses across different levels of HIV-associated immunosuppression.

HIV-1 viral load was quantified using the Xpert HIV-1 Viral Load Assay on the GeneXpert platform (Cepheid, Sunnyvale, CA, USA). This assay is an *in vitro* reverse transcriptase polymerase chain reaction (RT-PCR) test for the detection and quantification of HIV-1 RNA in human plasma. The test can quantify HIV-1 RNA over the range of 40 to 10,000,000 copies/mL and is validated for quantification of RNA from HIV-1 Group M (subtypes A, B, C, D, F, G, H, J, K, CRF01_AE, CRF02_AG, and CRF03_AB), Group N, and Group O. For viral load testing, plasma was separated from the EDTA whole blood samples by centrifugation at 1500 x g for 10 minutes at 4°C. One milliliter of plasma was then pipetted directly into the Xpert HIV-1 Viral Load cartridge. The GeneXpert System automated all steps of nucleic acid amplification testing, including sample preparation, nucleic acid extraction and amplification, and detection of the target sequence using real-time RT-PCR. Results were available within 90 minutes.

Viral loads were classified as detectable (≥40 copies/mL) or undetectable (<40 copies/mL) based on the assay’s lower limit of quantification. For analysis purposes, viral load results were log-transformed to normalize their distribution.

### 2.7 Statistical analyses

All statistical analyses were performed using IBM SPSS Statistics, Version 29.0 (IBM Corp., 2021). Descriptive statistics were calculated for demographic and clinical characteristics, stratified by CD4 count categories. Chi-square tests were used to assess associations between categorical variables.

To examine the relationship between CD4 counts and cytokine levels in HIV patients with and without CC, we conducted correlation analyses. Fisher’s Z-test was employed to compare the strength of these correlations between groups. Cytokine concentrations were log-transformed to normalize their distribution.

Multivariate regression analyses were performed to investigate the effects of CD4 counts, CC status, and potential confounding factors (age, sex, viral load, HIV stage, and ART duration) on cytokine levels. Interaction terms (CD4 count × cysticercosis status and sex × cysticercosis status) were included to assess potential effect modifications. Variance Inflation Factors (VIF) were calculated to check for multicollinearity among predictor variables.

Sensitivity analyses were conducted sequentially excluding each confounding factor to assess their impact on the relationship between CD4 counts and cytokine levels. Additionally, we performed stratified analyses by sex to explore potential sex-specific differences in cytokine predictors.

A two-way Multivariate Analysis of Variance (MANOVA) was conducted to examine differences in overall cytokine profiles across CD4 categories and CC status, as well as their interaction. Wilks’ Lambda, Pillai’s Trace, Hotelling-Lawley Trace, and Roy’s Greatest Root were used to assess the overall significance of the model. Partial eta squared (η²) values were calculated to estimate effect sizes. To account for multiple comparisons in the multivariate regression analyses (**Tables 1B, C**, **2**), we applied the Benjamini-Hochberg procedure to control the false discovery rate at 0.05 for all reported p-values.

**Table 1A:** A sensitivity analysis of CD4 count as a predictor for cytokine levels.

Cytokine	Confounder Excluded	β (CD4 count)	SE	p-value
TNF-a	None	-0.0012	0.000403	0.003
	Age	-0.0012	0.000403	0.003
	Sex	-0.0012	0.000403	0.003
	Viral Load	-0.0012	0.000403	0.003
	HIV Stage	-0.0012	0.000403	0.003
	ART Months	-0.0012	0.000403	0.003
	CC+	-0.0012	0.000403	0.003
	CD4_count_CC+	-0.0007	0.000303	0.004
	Sex_CC+	-0.0012	0.000403	0.003
IL-1β	None	-0.0007	0.000357	0.083
	Age	-0.0007	0.000357	0.083
	Sex	-0.0007	0.000357	0.083
	Viral Load	-0.0007	0.000357	0.083
	HIV Stage	-0.0007	0.000357	0.083
	ART Months	-0.0007	0.000357	0.083
	CC+	-0.0007	0.000357	0.083
	CD4_count_CC+	-0.0005	0.000357	0.123
	Sex_CC+	-0.0007	0.000357	0.083
IFN-γ	None	-0.001	0.0004	0.021
	Age	-0.001	0.0004	0.021
	Sex	-0.001	0.0004	0.021
	Viral Load	-0.001	0.0004	0.021
	HIV Stage	-0.001	0.0004	0.021
	ART Months	-0.001	0.0004	0.021
	CC+	-0.001	0.0004	0.021
	CD4_count_CC+	-0.0006	0.0003	0.088
	Sex_CC+	-0.001	0.0004	0.021

β, Regression coefficient representing the effect size of CD4 count on cytokine levels; SE, Standard Error of the regression coefficient; CD4_count × CC+, Interaction term between CD4 count and cysticercosis status; TNF-α, Tumor Necrosis Factor-alpha; IL-1β, Interleukin-1 beta; IFN-γ, Interferon-gamma. p < 0.05 indicates statistical significance.

Each confounding factor was sequentially excluded to assess its impact on the associations.

**Table 1B:** Multivariate regression analysis of cytokine levels in male HIV-positive patients.

Cytokine	Variables	β (SE)	95% CI	p-value	Adj p-value (BH)
TNF-a	CD4 count	-0.0017 (0.0006)	(-0.0030, -0.0005)	0.008	0.045
	CC+	-0.4029 (0.23)	(-0.8627, 0.0570)	0.085	0.199
	Age	-0.0081 (0.0077)	(-0.0235, 0.0074)	0.302	0.453
	Viral Load	-0.000002 (0.000002)	(-0.000005, 0.000002)	0.285	0.452
	HIV_Stage	0.2647 (0.0947)	(0.0754, 0.4541)	0.007	0.047
	ART Months	0.0002 (0.0021)	(-0.0039, 0.0043)	0.916	0.951
	Sex_CC+	-0.4029 (0.23)	(-0.8627, 0.0570)	0.085	0.199
	CD4_count_CC+	0.0022 (0.0009)	(0.0003, 0.0040)	0.026	0.118
IL-1β	CD4 count	-0.0012 (0.0007)	(-0.0026, 0.0002)	0.088	0.182
	CC+	-0.4779 (0.2567)	(-0.9912, 0.0354)	0.067	0.192
	Age	-0.0076 (0.0086)	(-0.0249, 0.0096)	0.379	0.539
	Viral Load	0.0000001 (0.000002)	(-0.000004, 0.000004)	0.944	0.944
	HIV_Stage	0.0584 (0.1057)	(-0.1529, 0.2697)	0.583	0.655
	ART Months	0.0015 (0.0023)	(-0.0031, 0.0061)	0.512	0.628
	Sex_CC+	-0.4779 (0.2567)	(-0.9912, 0.0354)	0.067	0.192
	CD4_count_CC+	0.0022 (0.0011)	(0.0001, 0.0043)	0.043	0.146
IFN-γ	CD4 count	-0.0014 (0.0007)	(-0.0027, -0.0001)	0.040	0.153
	CC+	-0.199 (0.2448)	(-0.6885, 0.2906)	0.420	0.553
	Age	-0.01 (0.0082)	(-0.0265, 0.0065)	0.230	0.388
	Viral Load	0.0000005 (0.000002)	(-0.000003, 0.000004)	0.795	0.858
	HIV_Stage	0.1431 (0.1008)	(-0.0584, 0.3447)	0.161	0.289
	ART Months	0.0014 (0.0022)	(-0.0030, 0.0058)	0.521	0.611
	Sex_CC+	-0.199 (0.2448)	(-0.6885, 0.2906)	0.420	0.553
	CD4_count_CC+	0.0015 (0.001)	(-0.0005, 0.0036)	0.129	0.248

β, Regression coefficient; SE, Standard Error; CI, Confidence Interval; CD4_count × CC+, Interaction term between CD4 count and cysticercosis status; TNF-α, Tumor Necrosis Factor-alpha; IL-1β, Interleukin-1 beta; IFN-γ, Interferon-gamma; ART, Antiretroviral Therapy; CC+, Cysticercosis-positive status.

Statistical Notes: Adjusted p-values were calculated using the Benjamini-Hochberg correction to control the false discovery rate at 0.05.

**Table 1C:** Multivariate regression analysis of cytokine levels in female HIV-positive patients.

Cytokine	Variables	β (SE)	95% CI	p-value	Adj p-value (BH)
TNF-a	CD4 count	-0.0014 (0.0005)	(-0.0027, -0.0001)	0.042	0.281
	CC+	-0.5632 (0.2938)	(-1.1545, 0.0281)	0.065	0.196
	Age	0.0011 (0.0108)	(-0.0204, 0.0227)	0.922	0.922
	Viral Load	-0.000009 (0.00001)	(-0.000030, 0.000012)	0.387	0.551
	HIV_Stage	-0.0948 (0.1391)	(-0.3777, 0.1881)	0.5	0.5870
	ART Months	-0.0025 (0.0031)	(-0.0089, 0.0038)	0.422	0.518
	Sex_CC+	-0.4929 (0.2466)	(-0.9945, 0.0088)	0.054	0.208
	CD4_count_CC+	0.0013 (0.0009)	(-0.0006, 0.0033)	0.180	0.348
IL-1β	CD4 count	-0.0004 (0.0005)	(-0.0013, 0.0006)	0.409	0.552
	CC+	-0.2179 (0.1032)	(-0.4277, -0.0080)	0.042	0.208
	Age	0.0074 (0.009)	(-0.0109, 0.0257)	0.418	0.538
	Viral Load	0.000013 (0.000009)	(-0.000005, 0.000031)	0.143	0.321
	HIV_Stage	0.016 (0.1164)	(-0.2208, 0.2527)	0.892	0.926
	ART Months	-0.005 (0.0026)	(-0.0103, 0.0003)	0.063	0.213
	Sex_CC+	-0.4357 (0.2063)	(-0.8555, -0.0159)	0.042	0.208
	CD4_count_CC+	0.0009 (0.0008)	(-0.0007, 0.0026)	0.256	0.406
IFN-γ	CD4 count	-0.0007 (0.0006)	(-0.0020, 0.0005)	0.252	0.424
	CC+	-0.215 (0.1343)	(-0.4883, 0.0583)	0.119	0.306
	Age	-0.0034 (0.0117)	(-0.0272, 0.0205)	0.776	0.838
	Viral Load	0.000012 (0.000011)	(-0.000011, 0.000035)	0.294	0.441
	HIV_Stage	0.0836 (0.1515)	(-0.2246, 0.3919)	0.585	0.658
	ART Months	-0.0048 (0.0034)	(-0.0117, 0.0021)	0.166	0.345
	Sex_CC+	-0.4301 (0.2687)	(-0.9767, 0.1166)	0.119	0.306
	CD4_count_CC+	0.0014 (0.001)	(-0.0007, 0.0035)	0.192	0.346

β, Regression coefficient; SE, Standard Error; CI, Confidence Interval; CD4_count × CC+, Interaction term between CD4 count and cysticercosis status and (Sex × CC+) interaction term between sex and cysticercosis status; TNF-α, Tumor Necrosis Factor-alpha; IL-1β, Interleukin-1 beta; IFN-γ, Interferon-gamma; ART, Antiretroviral Therapy; CC+, Cysticercosis-positive status.

Statistical Notes: Adjusted p-values were Benjamini-Hochberg corrected to control the false discovery rate at 0.05.

**Table 2:** MANOVA results for cytokine levels across CD4 categories and cysticercosis status.

Variable	Test Statistic	Value	F-value	p-value	Partial η²
Combined Cytokines	Λ	0.9159	1.41	0.1711	0.0841
	V	0.0841	1.41	0.1711	0.0841
	T²	0.0918	1.41	0.1711	0.0841
	θ	0.0918	1.41	0.1711	0.0841
CD4 Categories	Λ	0.9159	1.41	0.1711	0.0841
Cysticercosis	Λ	0.9082	1.51	0.128	0.0918
Interaction	Λ	0.915	1.43	0.1654	0.085

Wilks’ Lambda (Λ), Pillai’s Trace (V), Hotelling-Lawley Trace (T²), Roy’s Greatest Root (θ), f-statistics (f-value) and η² (eta squared) represents effect size. Small effect (η² ≈ 0.01), Medium effect (η² ≈ 0.06), Large effect (η² ≈ 0.14).”

For all analyses, a p-value < 0.05 was considered statistically significant. Where applicable, 95% confidence intervals were reported alongside effect estimates.

## 3 Results

### 3.1 Baseline characteristics of study participants

A total of 110 PLWHA individuals were included in this study. The median age of participants was 43 years (interquartile range [IQR]: 17 years). Participants were categorized into three CD4 count groups: normal (NIS) (>500 cells/mm³), moderate (MIS) (200-499 cells/mm³), and severe (SIM) (<200 cells/mm³). Most participants fell into the normal and moderate ranges, with 47 (43%) in the normal range and 54 (49%) in the moderate range. A smaller proportion, 9 (8%) of participants, were in the severe range. Significant differences were observed in the distribution of participants across CD4 categories based on region (p = 0.035) and sex (p = 0.020). Most participants were from Mbeya (70.0%), with a higher proportion in the moderate CD4 category (76%) compared to Iringa. Male participants predominated overall 69 (63%) but were overrepresented in the moderate category 38 (70%) and severe category 8 (89%).

Many participants had secondary education 63 (57%), were farmers 87 (79%), and were single 79 (72%). BMI distribution showed that most participants were of normal weight 75 (68%), with some variations across CD4 categories, though not statistically significant (p = 0.137) (**Table 3**).

**Table 3:** Demographics characteristics of the participants by CD4.

Variables	NIS	MIS	SIM	Total	P-value
Region					0.035
Iringa	14 (29.79%)	13 (24.07%)	6 (66.67%)	33 (30.00%)	
Mbeya	33 (70.21%)	41 (75.93%)	3 (33.33%)	77 (70.00%)	
Age Group					0.238
25 - 34	12 (25.53%)	7 (12.96%)	0 (0.00%)	19 (17.27%)	
35 - 49	19 (40.43%)	29 (53.70%)	6 (66.67%)	54 (49.09%)	
>50	16 (34.04%)	18 (33.33%)	3 (33.33%)	37 (33.64%)	
Sex					0.020
Male	23 (48.94%)	38 (70.37%)	8 (88.89%)	69 (62.73%)	
Female	24 (51.06%)	16 (29.63%)	1 (11.11%)	41 (37.27%)	
Edu_LvL					0.630
No formal Edu	6 (12.77%)	9 (16.67%)	1 (11.11%)	16 (14.55%)	
Primary Edu	9 (19.15%)	15 (27.78%)	2 (22.22%)	26 (23.64%)	
Secondary Edu	28 (59.57%)	29 (53.70%)	6 (66.67%)	63 (57.27%)	
Higher Edu	4 (8.51%)	1 (1.85%)	0 (0.00%)	5 (4.55%)	
Occupation					0.679
Farmer	36 (76.60%)	44 (81.48%)	7 (77.78%)	87 (79.09%)	
Employed	8 (17.02%)	9 (16.67%)	1 (11.11%)	18 (16.36%)	
Unemployed	3 (6.38%)	1 (1.85%)	1 (11.11%)	5 (4.55%)	
Marital Status					0.782
Single	33 (70.21%)	39 (72.22%)	7 (77.78%)	79 (71.82%)	
Married	4 (8.51%)	2 (3.70%)	0 (0.00%)	6 (5.45%)	
Prev. Married	10 (21.28%)	13 (24.07%)	2 (22.22%)	25 (22.73%)	
BMI					0.137
Underweight	4 (8.51%)	5 (9.26%)	0 (0.00%)	9 (8.18%)	
Normal weight	26 (55.32%)	41 (75.93%)	8 (88.89%)	75 (68.18%)	
Overweight	12 (25.53%)	5 (9.26%)	0 (0.00%)	17 (15.45%)	
Obese	5 (10.64%)	3 (5.56%)	1 (11.11%)	9 (8.18%)	

P-values were calculated using Chi-square test to assess associations between categorical variables. Statistical significance was defined as p < 0.05. Edu_LvL, Education Level; NIS, Normal Immune Status (>500 cells/mm³); MIS, Moderate Immunosuppression (200-499 cells/mm³); SIM, Severe Immunosuppression (<200 cells/mm³). CD4 count is measured in cells per mm³. All analyses were conducted using standard Chi-square assumptions without further correction.

Bold values indicate statistical significance at *p* < 0.05.

### 3.2 Serological and neuroradiological findings

Serological tests for CC revealed that all 110 participants underwent both *T. solium* (TsAb) antibody and *T. solium* (TsAg) antigen testing, while 94 participants also received cerebral CT imaging to assess for NCC. Of the total participants, 47 (43%) were found to be CC+, with 22 (20%) being diagnosed with NCC based on CT findings. Among the 47 CC+ participants, 46 were positive for TsAg. Of these, 33 (72%) were positive for TsAg alone, and 13 (28%) were positive for both TsAg and TsAb. Of the 33 who were TsAg positive alone, only 5 (15%) were also NCC positive. Additionally, 10 participants were positive for TsAb alone, with 5 of these (50%) also being NCC positive. The distribution of TsAb and TsAg positivity across CD4 categories showed no statistically significant differences (p = 0.7473 for TsAb; p = 0.4511 for TsAg) (**Table 4**). Among those with normal CD4 counts (>500 cells/mm³), 10 (21%) were TsAb positive and 21(45%) were TsAg positive, compared to 12 (22%) and 23(43%) respectively in the moderate CD4 group (200-499 cells/mm³), and 1 (11%) and 2 (22%) in the severe CD4 group (<200 cells/mm³).

**Table 4:** Distribution of clinical characteristics by CD4 categories.

Variable	NIS	MIS	SIM	Total	P-value
TsAb					0.747
Negative	37 (78.72%)	42 (77.78%)	8 (88.89%)	87 (79.09%)	
Positive	10 (21.28%)	12 (22.22%)	1 (11.11%)	23 (20.91%)	
TsAg					0.451
Negative	26 (55.32%)	31 (57.41%)	7 (77.78%)	64 (58.18%)	
Positive	21 (44.68%)	23 (42.59%)	2 (22.22%)	46 (41.82%)	
CC status					0.026
Negative	25 (53.19%)	29 (53.70%)	9 (100.00%)	63 (57.27%)	
Positive	22 (46.81%)	25 (46.30%)	0 (0.00%)	47 (42.73%)	
NCC status					0.430
No cyst	29 (74.36%)	35 (76.09%)	8 (88.89%)	72 (76.60%)	
Active	5 (12.82%)	3 (6.52%)	1 (11.11%)	9 (9.57%)	
Inactive	5 (12.82%)	8 (17.39%)	0 (0.00%)	13 (13.83%)	
Cyst stage					0.575
No Cysts	29 (74.36%)	35 (76.09%)	8 (88.89%)	72 (76.60%)	
Vesicular	3 (7.69%)	2 (4.35%)	0 (0.00%)	5 (5.32%)	
Calcified	5 (12.82%)	8 (17.39%)	0 (0.00%)	13 (13.83%)	
Mixed	2 (5.13%)	1 (2.17%)	1 (11.11%)	4 (4.26%)	
No. of Cysts					0.452
No Cyst	29 (74.36%)	35 (76.09%)	8 (88.89%)	72 (76.60%)	
Single	3 (7.69%)	1 (2.17%)	0 (0.00%)	4 (4.26%)	
Multiple	7 (17.95%)	10 (21.74%)	1 (11.11%)	18 (19.15%)	

P-values were calculated using the Chi-square test to assess associations between categorical clinical variables across CD4 categories. Statistical significance was determined at p < 0.05. TsAb, Taenia solium Antibody; TsAg, Taenia solium Antigen; CC+, Cysticercosis positive; NIS, Normal Immune Status (>500 cells/mm³); MIS, Moderate Immunosuppression (200-499 cells/mm³); SIM, Severe Immunosuppression (<200 cells/mm³).

There was a statistically significant difference in the distribution of CC positivity across CD4 categories (p = 0.026). Notably, no participants in the severe CD4 group were positive for CC, while 22 (47%) and 25 (46%) were positive in the normal and moderate CD4 groups, respectively (**Table 4**).

Of the 94 HIV-positive participants who underwent cerebral CT imaging, 22 (23%) showed brain cysts indicative of NCC, with 9 (10%) showing active cysts and 13 (14%) showing inactive cysts. The distribution of NCC status across CD4 categories did not show statistically significant differences (p = 0.430) (**Table 4**).

Cyst stages varied across CD4 categories, but these differences were not statistically significant (p = 0.575). Vesicular cysts were found in 3 (8%) of the normal CD4 group, 2 (4%) of the moderate CD4 group, and none in the severe CD4 group. Calcified cysts were observed in 5 (13%), and 8 (17%), of the normal and moderate groups respectively; none in the severe CD4 group. Mixed-stage cysts were present in 1 (5%) of the normal CD4 group, 1 (2%) of the moderate CD4 group, and 1 (11%) of the severe CD4 group (**Table 4**).

The number of cysts also varied across CD4 categories, but these differences were not statistically significant (p = 0.452). Multiple cysts were found in 7 (18%) of the normal CD4 group, 10 (22%) of the moderate CD4 group, and 1 (11%) of the severe CD4 group. Single cysts were observed in 3 (8%) of the normal CD4 group, 1 (2%) of the moderate, and none of the severe CD4 group (**Table 4**).

### 3.3 Cytokine profiles by CD4 categories

**Figure 1:**
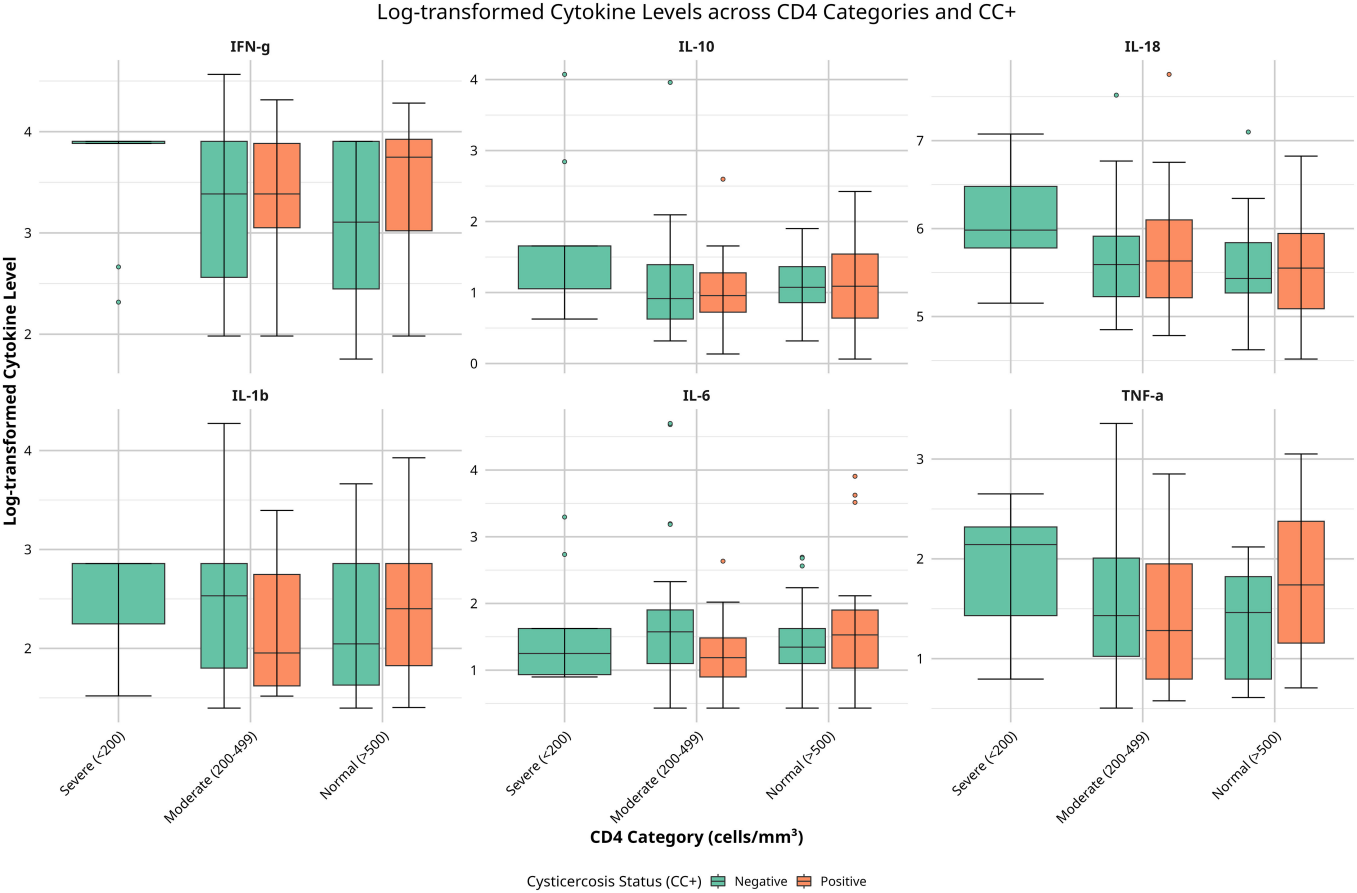
Boxplots of serum cytokine levels (TNF-α, IL-6, IL-10, IL-1β, IFN-γ, IL-18) across CD4 categories (<200, 200–499, ≥500 cells/mm³) and cysticercosis status (positive or negative). Colors indicate cysticercosis status (orange: positive; green: negative). Data were log-transformed. Significant differences (p < 0.05) by Kruskal-Wallis tests are noted.

Cytokine levels revealed complex patterns across CD4 categories and CC status. Notably, there were no CC+ individuals in the severe CD4 group (<200 cells/mm³), which restricted comparisons to CC- individuals only in this category. Among CC- individuals, TNF-α, IL-18, and IL-1β levels were elevated in the severe immunosuppression (SIM) group compared to the moderate (MIS) and normal (NIS) groups, suggesting increased immune activation associated with severe immunosuppression. In contrast, in CC+ individuals, TNF-α and IL-1β levels were higher in the normal immune status (NIS) group compared to the moderate immunosuppression (MIS) group, suggesting that CC may influence cytokine regulation differently based on CD4 status (**Figure 1**).

IL-10 showed a relatively consistent pattern across CD4 categories for CC- individuals, while CC+ individuals in the normal CD4 group exhibited a wider range of IL-10 levels, suggesting a varied regulatory response (**Figure 1**). IL-18 levels were higher and more variable in CC- participants across all CD4 categories compared to CC+ individuals, suggesting a distinct immune modulation due to CC. No significant changes were observed for IL-6, IL-1β (in CC+ individuals), and IFN-γ across CD4 categories and CC status. In summary, these findings reveal differences in cytokine levels based on CD4 count and CC status, particularly with elevated TNF-α, IL-18, and IL-1β in CC- individuals with severe immunosuppression. This suggests that immune responses may vary depending on CD4 count and CC infection in HIV-positive individuals.

### 3.4 Correlation between CD4 counts, cytokine levels, and cysticercosis status

**Figure 2:**
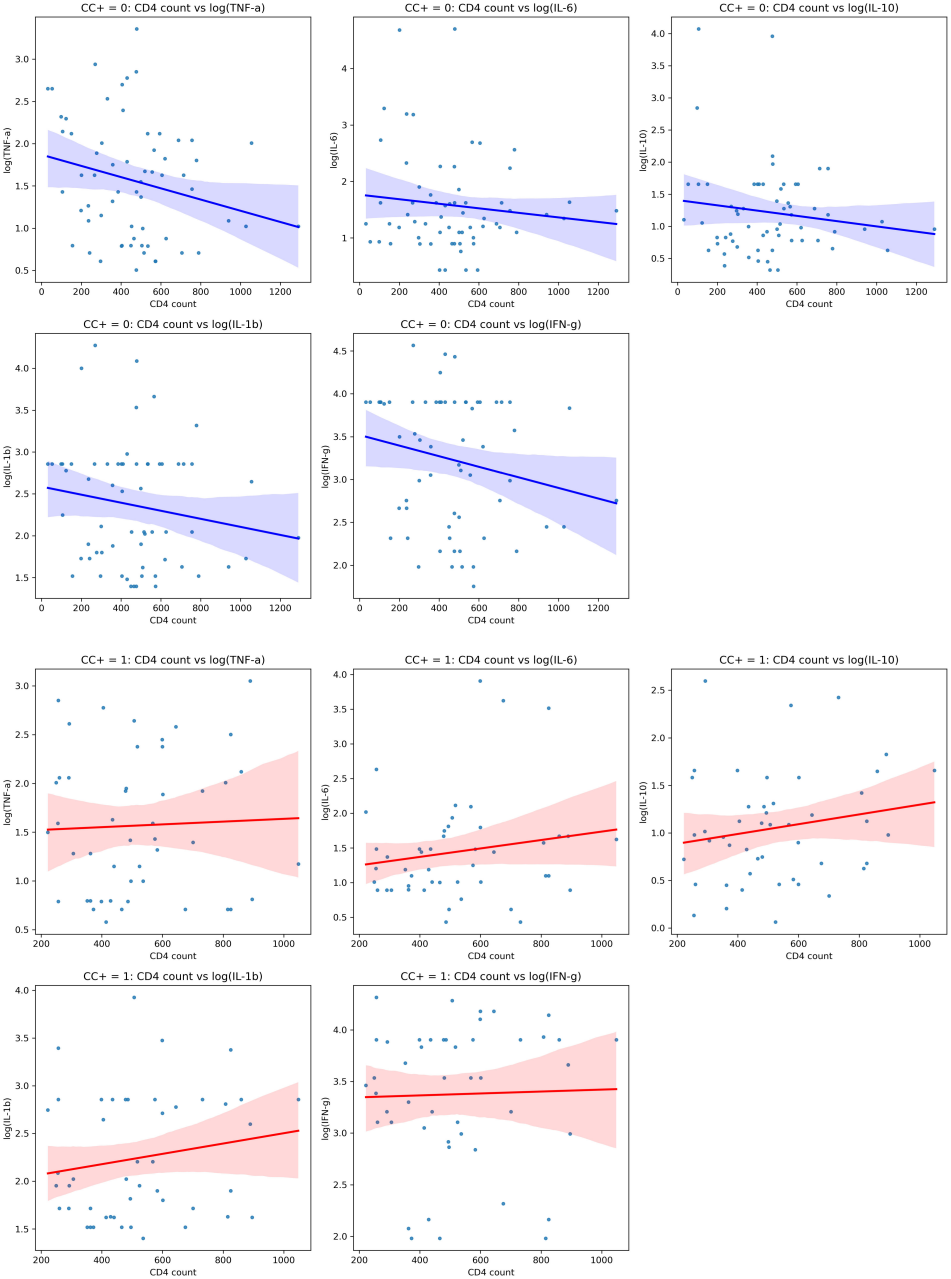
Scatterplots showing correlations between CD4 counts and cytokine levels (TNF-α, IL-6, IL-10, IL-1β, IFN-γ)in individuals with different cysticercosis (CC) statuses. The first panel (top) represents CC-negative individuals (blue), displaying weak negative correlations. The second panel (bottom) represents CC-positive individuals (red), showing weak positive correlations. The shaded areas indicate the 95% confidence intervals. Spearman’s rank correlation coefficients (r) are provided for each plot; statistical significance is indicated where p < 0.05.

**Figure 2** shows the relationships between CD4 counts and cytokine levels in HIV-positive individuals, both with and without CC. We conducted correlation analyses to explore the associations between CD4 counts, cytokine levels, and CC status. This helped us understand whether group-level trends are reflected at the individual level and provided deeper insights into how immune function (cytokine responses) might be influenced by both HIV and CC (**Figure 2**).

In CC-negative individuals, we observed weak negative correlations between CD4 counts and several cytokines, including TNF-α (r = –0.236, p = 0.061), IL-6 (r = –0.115, p = 0.362), IL-1β (r = –0.166, p = 0.195), IFN-γ (r = –0.200, p = 0.109), and the regulatory cytokine IL-10 (r = –0.144, p = 0.260). TNF-α, IL-6, IL-1β, IFN-γ, and IL-10. Although these correlations were not statistically significant, the trend suggested that higher CD4 counts might be linked to lower levels of these pro-inflammatory cytokines (**Figure 2A**).

In contrast, in CC-positive individuals, the correlations between CD4 counts and cytokine levels were generally positive for TNF-α (r = 0.041, p = 0.772), IL-6 (r = 0.166, p = 0.269), IL-10 (r = 0.181, p = 0.226), IL-1β (r = 0.170, p = 0.255), and IFN-γ (r = 0.028, p = 0.854), though none of these were statistically significant either. The positive trends may indicate a different immune response in the presence of CC (**Figure 2B**).

Although these results did not reach statistical significance, Fisher’s Z-test indicated that the differences in correlation strength between CC-positive and CC-negative individuals for IL-10 (Z-diff = 1.655, p = 0.098) and IL-1β (Z-diff = 1.705, p = 0.088) were close to significant, suggesting there may be differences in how these cytokines are regulated depending on CC status.

In summary, these findings suggest that cytokine levels may correlate to CD4 counts in different ways depending on whether the individual has CC. While the correlations were not statistically significant, the trends observed imply that CC might affect immune responses in HIV-positive individuals, especially regarding pro-inflammatory and regulatory cytokines like IL-10 and IL-1β.

### 3.5 Multivariate analysis of cytokine predictors

To further investigate the relationships among CD4 counts, cytokine levels, and CC status, we performed a multivariate regression analysis. This analysis was adjusted for potential confounding factors such as age, sex, viral load, HIV stage, and duration on ART. This approach allows us to assess the independent effects of each factor on cytokine levels in HIV-positive individuals with and without CC. To account for multiple comparisons and control the false discovery rate at 0.05, we applied the Benjamini-Hochberg procedure (**Table 5**).

**Table 5:** Multivariate regression analysis of cytokine levels in relation to CD4 count, cysticercosis status, and other confounding factors.

Cytokine	Variables	β (SE)	95% CI	p-value	Adj p-value (BH)
**TNF-a**	CD4 count	-0.0012 (0.0004)	(-0.0020, -0.0004)	0.003	**0.028**
	CC+	0.1450 (0.4751)	(-0.7976, 1.0876)	0.761	0.815
	Age	-0.0083 (0.0063)	(-0.0207, 0.0041)	0.188	0.452
	Sex	0.3764 (0.1956)	(-0.0117, 0.7645)	0.057	0.245
	Viral Load	-0.0000 (0.0000)	(-0.0000, 0.0000)	0.296	0.572
	HIV_Stage	0.1486 (0.0760)	(-0.0021, 0.2993)	0.053	0.266
	ART Months	-0.0009 (0.0017)	(-0.0043, 0.0025)	0.596	0.813
	Sex_CC+	-0.6666 (0.3022)	(-1.2662, -0.0671)	0.030	0.178
	CD4_count_CC+	0.0016 (0.0007)	(0.0003, 0.0029)	0.018	0.137
**IL-6**	CD4 count	-0.0005 (0.0005)	(-0.0014, 0.0005)	0.347	0.613
	CC+	-0.3694 (0.5707)	(-1.5016, 0.7628)	0.519	0.759
	Age	0.0022 (0.0075)	(-0.0127, 0.0172)	0.768	0.809
	Sex	-0.1258 (0.2350)	(-0.5920, 0.3404)	0.594	0.828
	Viral Load	-0.0000 (0.0000)	(-0.0000, 0.0000)	0.859	0.889
	HIV_Stage	0.0696 (0.0912)	(-0.1115, 0.2506)	0.448	0.746
	ART Months	0.0014 (0.0020)	(-0.0027, 0.0054)	0.506	0.759
	Sex_CC+	-0.3664 (0.3630)	(-1.0866, 0.3538)	0.315	0.573
	CD4_count_CC+	0.0014 (0.0008)	(-0.0001, 0.0030)	0.079	0.273
**IL-10**	CD4 count	-0.0005 (0.0004)	(-0.0013, 0.0003)	0.193	0.429
	CC+	-0.3113 (0.4562)	(-1.2163, 0.5938)	0.497	0.784
	Age	0.0006 (0.0060)	(-0.0114, 0.0125)	0.922	0.937
	Sex	0.2856 (0.1878)	(-0.0871, 0.6582)	0.1316	0.376
	Viral Load	0.0000 (0.0000)	(-0.0000, 0.0000)	0.693	0.785
	HIV_Stage	0.0331 (0.0729)	(-0.1116, 0.1777)	0.651	0.766
	ART Months	-0.0031 (0.0016)	(-0.0063, 0.0001)	0.059	0.236
	Sex_CC+	-0.1955 (0.2902)	(-0.7712, 0.3802)	0.502	0.772
	CD4_count_CC+	0.0008 (0.0006)	(-0.0004, 0.0021)	0.1905	0.440
**IL-1β**	CD4 count	-0.0007 (0.0004)	(-0.0015, 0.0001)	0.083	0.276
	CC+	-0.0135 (0.4840)	(-0.9738, 0.9467)	0.978	0.978
	Age	-0.0021 (0.0064)	(-0.0148, 0.0105)	0.738	0.805
	Sex	0.3018 (0.1993)	(-0.0936, 0.6972)	0.133	0.363
	Viral Load	0.0000 (0.0000)	(-0.0000, 0.0000)	0.658	0.760
	HIV_Stage	0.0441 (0.0774)	(-0.1094, 0.1976)	0.570	0.814
	ART Months	-0.0009 (0.0017)	(-0.0043, 0.0026)	0.621	0.793
	Sex_CC+	-0.5947 (0.3079)	(-1.2055, 0.0161)	0.056	0.259
	CD4_count_CC+	0.0014 (0.0007)	(0.0001, 0.0028)	0.036	0.194
**IFN**-γ	CD4 count	-0.0010 (0.0004)	(-0.0018, -0.0002)	0.021	0.142
	CC+	0.2650 (0.5026)	(-0.7321, 1.2622)	0.599	0.799
	Age	-0.0072 (0.0066)	(-0.0204, 0.0060)	0.281	0.562
	Sex	0.3031 (0.2069)	(-0.1075, 0.7136)	0.146	0.381
	Viral Load	0.0000 (0.0000)	(-0.0000, 0.0000)	0.601	0.784
	HIV_Stage	0.1306 (0.0803)	(-0.0288, 0.2900)	0.107	0.321
	ART Months	-0.0009 (0.0018)	(-0.0044, 0.0027)	0.627	0.767
	Sex_CC+	-0.5285 (0.3197)	(-1.1627, 0.1058)	0.102	0.321
	CD4_count_CC+	0.0013 (0.0007)	(-0.0001, 0.0027)	0.074	0.260
**IL-18**	CD4 count	-0.0005 (0.0004)	(-0.0012, 0.0002)	0.185	0.463
	CC+	0.5246 (0.4227)	(-0.3140, 1.3633)	0.218	0.466
	Age	-0.0038 (0.0056)	(-0.0149, 0.0072)	0.494	0.801
	Sex	0.0622 (0.1741)	(-0.2831, 0.4075)	0.722	0.802
	Viral Load	-0.0000 (0.0000)	(-0.0000, 0.0000)	0.626	0.782
	HIV_Stage	0.0566 (0.0676)	(-0.0775, 0.1907)	0.404	0.693
	ART Months	-0.0018 (0.0015)	(-0.0048, 0.0011)	0.224	0.463
	Sex_CC+	-0.2738 (0.2689)	(-0.8073, 0.2596)	0.311	0.583
	CD4_count_CC+	-0.0003 (0.0006)	(-0.0014, 0.0009)	0.636	0.763

Multivariate regression analysis of cytokine predictors of CD4 count in HIV-positive individuals with and without cysticercosis, adjusted for age, sex, viral load, HIV stage, and duration on ART. cysticercosis status (CC+), their interaction terms (CD4 count × CC+), and (Sex × CC+). Regression coefficients (β), standard errors (SE), and Adj p-value BH - Benjamin Hochberg adjusted p-value.

Bold values indicate statistical significance at *p* < 0.05.

After adjustment, several significant associations emerged. TNF-α levels were negatively associated with CD4 count (β = –0.0012, 95% CI: –0.0020 to –0.0004, p = 0.003), meaning lower CD4 counts were linked to higher TNF-α levels. Additionally, there was a significant positive interaction between CD4 count and CC status (β = 0.0016, 95% CI: 0.0003 to 0.0029, p = 0.018), suggesting that CC may alter the effect of CD4 count on TNF-α levels. IFN-γ levels also showed a significant negative association with CD4 count (β = –0.0010, 95% CI: –0.0018 to –0.0002, p = 0.021). The interaction between sex and CC status was significant for TNF-α (β = –0.6666, 95% CI: –1.2662 to –0.0671, p = 0.030), indicating possible sex-based differences in immune response.

Other associations, such as those between IL-1β and CD4 count (β = –0.0007, 95% CI: –0.0015 to 0.0001, p = 0.083), or interactions involving IL-1β and CC status (β = 0.0014, 95% CI: 0.0001 to 0.0028, p = 0.036) or IFN-γ and CC status (β = 0.0013, 95% CI: –0.0001 to 0.0027, p = 0.074) did not remain statistically significant after adjustment. No significant associations were found for IL-6, IL-10, or IL-18 in relation to CD4 count or CC status (all p-values > 0.05) (**Table 5**). These findings highlight the complexity of cytokine regulation in HIV-positive individuals with CC and suggest that CC might affect immune function differently depending on a person’s immune status.

### 3.6 Sensitivity and sex-stratified analyses of confounding factors

To ensure our multivariate regression findings were robust, we performed a sensitivity analysis to test whether individual confounding factors (age, sex, viral load, HIV stage, and ART duration) influenced the associations between cytokine levels and CD4 counts. We assessed how removing each confounder affected our results, and also checked for any sex-specific differences in these relationships. Before the sensitivity analysis, we confirmed that there was no multicollinearity between confounders. All variables exhibited low variance inflation factors (VIFs), with the highest being 1.267 for duration on ART (**Supplementary Table S2**), indicating that multicollinearity was not a concern in our multivariate regression models.

In the sensitivity analysis, we sequentially excluded each confounding factor from the model and observed the impact on the associations between cytokine levels and CD4 counts. For TNF-α, the association with CD4 count remained significant when excluding most confounders (β = –0.0012, 95% CI: –0.0020 to –0.0004, p = 0.003). However, when the CD4 count × cysticercosis status interaction term was removed, the association slightly changed but remained significant (β = –0.0007, 95% CI: –0.0013 to –0.0001, p = 0.004) (**Table 1A**).

For IFN-γ, the association with CD4 count remained significant across most exclusions (β = –0.0010, 95% CI: –0.0018 to –0.0002, p = 0.021) but became non-significant when excluding the CD4 count × cysticercosis status interaction term (β = –0.0006, 95% CI: –0.0012 to 0.0000, p = 0.088). This suggests that the interaction between CD4 count and CC status plays a critical role in the association with IFN-γ levels (**Table 1A**).

#### 3.6.1 Sex-stratified analysis

We also performed separate analyses for males and females, applying the Benjamini-Hochberg correction, to explore potential sex-specific differences in the associations between cytokine levels and CD4 counts. This investigates whether the patterns observed in the overall population persisted within each sex and to identify any differential effects that might inform sex-specific immune response dynamics (**Tables 1B**, **C**).

In males, significant predictors for TNF-α after correction were CD4 count and HIV stage. Higher CD4 counts were associated with lower TNF-α levels (β = –0.0017, 95% CI: –0.0030 to –0.0005, p = 0.045), while more advanced HIV stages were linked to higher TNF-α levels (β = 0.2647, 95% CI: 0.0754 to 0.4541, p = 0.047). However, the interaction between CD4 count and CC (β = 0.0022, 95% CI: 0.0003 to 0.0040, p = 0.118) was no longer significant after correction, implying that the interaction effect observed in the overall analysis was not pronounced in males (**Table 1B**). No significant predictors were found for IL-1β or IFN-γ after correction in males. These results suggest that TNF-α is influenced by both immune status (CD4 count) and disease progression (HIV stage) in males.

In females, after applying the Benjamini-Hochberg correction, no significant predictors were found for TNF-α, IL-1β, or IFN-γ (all adjusted p > 0.05). The associations that were previously significant before correction—for example, CD4 count with TNF-α (β = –0.0014, 95% CI: –0.0027 to –0.0001, p = 0.281), CC status with IL-1β (β = –0.2179, 95% CI: –0.4277 to –0.0080, p = 0.208), and the interaction between sex and CC status with IL-1β (β = –0.4357, 95% CI: –0.8555 to –0.0159, p = 0.208)—were no longer significant after correction. This suggests that in females, the associations between cytokine levels and CD4 counts are less pronounced or may be influenced by other factors not captured in our analysis (**Table 1C**).

Overall, the sensitivity analysis confirmed that the associations between cytokine levels and CD4 counts were generally robust, particularly for TNF-α. For IFN-γ, the association remained significant unless the interaction with CC status was excluded. The sex-stratified analysis showed that in males, TNF-α was significantly associated with both CD4 count and HIV stage, while no significant predictors were found in females after correction. These findings suggest that cytokine responses may vary between males and females, with males showing stronger associations between immune status and cytokine levels.

### 3.7 MANOVA analysis of overall cytokine profiles

To understand how CD4 categories and CC status together influence immune responses, we used a MANOVA to examine their effects on overall cytokine profiles. This method allowed us to analyze multiple cytokines at once to see if there were any significant differences across CD4 categories and CC status. The cytokines analyzed were TNF-α, IL-6, IL-10, IL-1β, IFN-γ, and IL-18. The results showed no significant effects for CD4 categories (Wilks’ Lambda = 0.9159, F(12, 276) = 1.41, p = 0.1711, partial η² = 0.0841), CC status (Wilks’ Lambda = 0.9082, F(6, 138) = 1.51, p = 0.1280, partial η² = 0.0918), or the interaction between CD4 categories and CC status (Wilks’ Lambda = 0.9150, F(12, 276) = 1.43, p = 0.1654, partial η² = 0.0850) on the combined cytokine profiles (TNF-a, IL-6, IL-10, IL-1β, IFN-g, and IL-18) (**Table 2**). Other multivariate test criteria (Pillai’s Trace, Hotelling-Lawley Trace, and Roy’s Greatest Root) gave similar results, all yielding identical F-values and p-values to those obtained with Wilks’ Lambda for each effect, confirming these findings (**Table 2**).

Although none of the effects reached statistical significance (p < 0.05), the effect sizes (partial η²) suggested small to moderate contributions. CC status accounted for 9.18%of the variance in cytokine levels, followed by the interaction between CD4 categories and CC status (8.50%), and CD4 categories alone (8.41%) (**Table 2**).

In conclusion, the MANOVA did not identify any statistically significant effects of CD4 count categories, CC status, or their interaction on the overall cytokine profiles. This suggests that, when considered collectively, neither CD4 count, nor CC significantly influenced the immune response in this HIV-positive population.

## 4 Discussion

The primary aim of this study was to explore whether co-infection with *T. solium*, a potent immunomodulator, alters immune regulation in HIV-positive individuals, particularly by influencing cytokine profiles and CD4+ T-cell counts, in Tanzania’s southern highlands. Our findings revealed that co-infection with *T. solium* significantly influenced immune responses, with TNF-α levels showing a strong negative association with CD4 counts, while this association was positively modulated by CC status. IFN-γ also displayed a significant negative relationship with CD4 counts, suggesting that *T. solium* co-infection may potentially alter immune regulation in HIV-positive individuals. While cytokines such as IL-1β, IL-6, IL-10, and IL-18, showed trends of immune modulation, these were not statistically significant.

Our sex-stratified analysis revealed important differences in immune regulation between male and female participants. In males, we found a significant negative association between TNF-α levels and CD4 counts, and a strong correlation between advanced HIV stages and elevated TNF-α levels. In contrast, females showed no significant associations between cytokine levels and CD4 counts after correction for multiple comparisons, suggesting more tightly regulated immune responses.

These sex-based differences align with previous research showing that testosterone typically suppresses both innate and adaptive immune responses, while estrogen enhances immune regulation (40). Female patients have been shown to exhibit stronger alterations in mucosal T-cell repertoire, with increased frequencies of Th1, Th17, and Th1/Th17-cell subsets compared to male patients (40, 41), which may explain our observation of fewer significant associations between CD4 counts and cytokine levels in the female cohort. Furthermore, female patients co-infected with HIV and helminths have been shown to exhibit better immunological recovery on ART compared to males (42), supporting our observation of more regulated immune responses in females. These findings suggest that sex-specific factors should be considered in developing therapeutic strategies for HIV-cysticercosis co-infection (43), with male patients potentially benefiting from modified anti-inflammatory interventions. Overall, these results highlight the potential impact of *T. solium* on immune function in PLWHA individuals, particularly in modulating pro-inflammatory and regulatory cytokine responses.

Similar patterns of immune dysregulation have been observed in other HIV-parasitic co-infections, such as schistosomiasis and soil-transmitted helminths, where elevated TNF-α and IL-6 persist, even in individuals on ART, indicating persistent immune activation (44–46). This occurs because helminths, including *T. solium*, release immunomodulatory molecules that drive a shift from a Th1 to a Th2 immune response, suppressing key pro-inflammatory cytokines like IFN-γ, while increasing IL-10 and IL-6, leading to continued but modulated immune activation (24, 25). In our study, this modulation was evident as *T. solium* co-infection altered the typical relationship between TNF-α and CD4 counts. While lower CD4 counts generally lead to increased TNF-α, the presence of CC moderated this increase, suggesting that the parasite’s immunomodulatory effects dampened the expected pro-inflammatory response, even in the face of advanced immunosuppression. This highlights the need for targeted immunomodulatory interventions to manage immune activation in these patients (47).

Our findings suggest that the cytokines TNF-α, IL-1β, and IFN-γ are central to the immune response in HIV-CC co-infection whereas others like IL-18 are not. The elevated levels of these pro-inflammatory cytokines in CC-positive individuals with preserved immune function (with CD4^+^ counts above 500 cells/µL) indicate an activated pro-inflammatory state, potentially driven by persistent antigenic stimulation from the parasite (48, 49). This continuous stimulation may modulate the balance between pro-inflammatory and regulatory cytokines, such as IL-10, exacerbating immune dysregulation despite partial immune recovery (50, 51). Similar patterns have been observed in studies where parasitic infections in HIV patients led to heightened immune activation, potentially exacerbating HIV progression (18, 52, 53). This persistent antigenic stimulation suggests that immune recovery, as measured by CD4^+^ counts, may not fully mitigate ongoing inflammation and the clinical consequences thereof in such contexts (54, 55).

An unexpected finding in our study was the lack of significant differences in cytokine levels across CD4^+^ categories based on individual cytokine analyses (e.g., TNF-α, IL-6, IL-10) and the absence of significant multivariate effects from the MANOVA, which assessed combined cytokine profiles across CD4^+^ categories, CC status, and their interaction. This suggests that, while immune activation occurs in co-infected individuals, it does not lead to widespread alterations in cytokine profiles across the PLWHA population. Also, when considering all cytokines simultaneously, there are no clear distinctions based on CD4^+^ categories or CC status alone. This finding supports the notion that immune modulation due to parasitic co-infections is targeted rather than systemic (49, 56), and indicates that the relationship between HIV, CC, and immune responses is more complex than initially hypothesized.

Comparative studies in HIV-negative individuals have reported varying immune responses to CC often with more regulated immune responses and less pronounced inflammation (25, 57). Our findings suggests that HIV infection alters this CC-driven immunological landscape, particularly in patients with low CD4 counts (SIM), leading to heightened pro-inflammatory responses such as elevated TNF-α and IL-18 levels. This aligns with studies indicating that PLWHA individuals may experience more severe manifestations of parasitic infections due to immune dysregulation (27, 58). While our study focused on CC, similar interactions have been observed with other helminth infections in PLWHA populations. Co-infections with helminths such as schistosomiasis, soil-transmitted helminths and filarial infections (*Wuchereria bancrofti, Onchocerca volvulus, Loa loa*) have been linked to increased immune activation, and changes in cytokine profiles, including elevated IL-10 and TNF-α, which can exacerbate immune suppression and impact HIV disease progression (24, 25, 59). These effects are often contingent on CD4^+^ counts, where severe immunosuppression is associated with greater immune dysregulation (elevated TNF-α and IL-18 levels, lower IFN-γ levels) (60, 61).

Several limitations of our study should be acknowledged. The sample size of 110 PLWHA patients may have limited our ability to detect certain associations, particularly in subgroup analyses. The cross-sectional design provides only a snapshot of immune status and cannot capture the dynamic nature of immune responses over time. Another limitation is the absence of an HIV-negative control group. However, previous studies have indicated that, under effective ART, cytokine profiles such as of IL-6, TNF and IL-1β of treated PLWHA individuals often closely resemble those of HIV-negative individuals (20). However, the primary goal of this study was to investigate the impact of CC on cytokines in relation to the CD4 counts in HIV positive individuals.

Despite these limitations, our study provides valuable insights into the complex interactions between HIV, CC, and immune responses. The observed persistence of elevated pro-inflammatory cytokines in CC-positive patients, along with the potential for differential immune responses based on CD4^+^ counts, highlights the need for more nuanced and personalized approaches to HIV care in endemic areas.

Future research should aim to address the limitations of the current study and build upon its findings. Longitudinal studies with larger cohorts, including both PLWHA and HIV-negative individuals, could provide a more comprehensive understanding of the dynamic interplay between HIV, CC and other common helminths and immune responses over time. Such studies could also help elucidate the long-term clinical implications of the observed immune dysregulation in co-infected individuals such as on vaccine efficacy.

Furthermore, exploring the impact of antiparasitic treatment on immune responses and HIV disease progression in co-infected individuals could inform more effective treatment strategies. The potential use of cytokine profiles as biomarkers for CC in HIV patients warrants further investigation. If validated, such biomarkers could aid in the early detection and monitoring of CC in PLWHA individuals, potentially improving outcomes through timely intervention.

## 5 Conclusion

In conclusion, our study highlights the complex interactions between HIV infection, CC, and immune responses. Although we observed elevated levels of pro-inflammatory cytokines—such as TNF-α, IL-1β, and IFN-γ—in CC-positive patients with higher CD4^+^ T-cell counts, these findings did not reach statistical significance. This suggests a trend toward immune activation that warrants further investigation. The lack of significant associations underscores the complexity of immune interactions in co-infected individuals and emphasizes the need for larger, longitudinal studies to better understand these dynamics. Considering parasitic co-infections and cytokine profiles in relation to the CD4 count in HIV management remains important, especially in endemic regions, to develop more targeted interventions that could improve patient outcomes.

## Data Availability

The raw data supporting the conclusions of this article will be made available by the authors, without undue reservation.
