# Supplementary material for: Cytokine profiles and CD4 counts in HIV-positive individuals with cysticercosis: implications for sex-specific immune responses in co-endemic regions of Tanzania
Source: Front Immunol. 2025 Feb 19;16:1521295. doi: 10.3389/fimmu.2025.1521295 (PMC11880214; doi:10.3389/fimmu.2025.1521295)
Supplement: Supplementary file 1 [file Table1.docx]

**Supplementary Data Analysis:**

To further explore the potential role of Body Mass Index (BMI) in modulating immune profiles, we conducted additional analyses examining the relationship between cytokine levels and CD4+ count across BMI categories. These analyses included multivariate regression models with interaction terms (CD4+ count × BMI) to assess potential effect modifications and stratified analyses to explore trends within specific BMI subgroups (underweight, normal weight, overweight, and obese).

**Table S1** presents the results of the multivariate regression models, highlighting the coefficients, confidence intervals, and p-values for cytokines of interest. Interaction terms were included to assess whether BMI modifies the relationship between CD4+ count and cytokine levels.

**Table S2** presents the stratified analysis of cytokine levels across BMI and CD4+ count categories, offering detailed insights into immune response variability within these subgroups.

**Table S3 presents the results of the multivariate regression models. Interaction terms were included to evaluate whether viral load modifies the relationship between sex and cytokine levels.**

**Multivariate Regression and Interaction Analysis:**

**Table 1: Multivariate Regression and Interaction Analysis of BMI and CD4+ Categories on Cytokine Levels**

| **Cytokine** | **variable** | **β (SE)** | **95% CI** | **p-value** | p-value (BH) |
| --- | --- | --- | --- | --- | --- |
| **TNF-a** | Underweight | -1.8289(2.823372) | (-7.43477, 3.776975) | 0.519 | 0.944 |
|  | Overweight | -0.69852(1.83881) | (-4.34952, 2.952482) | 0.705 | 0.963 |
|  | Obese | -1.94102(2.59366) | (-7.09079, 3.208752) | 0.456 | 0.889 |
|  | SIS | 2.884087(2.29182) | (-1.66638, 7.43455) | 0.211 | 0.74 |
|  | MIS | 0.791483(1.303903) | (-1.79745, 3.380413) | 0.545 | 0.95 |
|  | Underweight X MIS | 1.216505(3.82735) | (-6.38279, 8.815799) | 0.751 | 0.963 |
|  | Overweight X SIS | 0((empty)) | (, ) |  |  |
|  | Overweight X MIS | -0.0702(3.214672) | (-6.45301, 6.31261) | 0.983 | 0.983 |
|  | Obese X SIS | 2.451453(6.102639) | (-9.66548, 14.56839) | 0.689 | 0.963 |
|  | Obese X MIS | -0.3047(3.991894) | (-8.2307, 7.621298) | 0.939 | 0.983 |
|  | Age | -0.04882(0.048995) | (-0.1461, 0.048465) | 0.322 | 0.84 |
|  | Sex | 0.437351(1.095775) | (-1.73834, 2.613038) | 0.691 | 0.963 |
|  | CC+ | 0.335651(1.066242) | (-1.7814, 2.4527) | 0.754 | 0.963 |
|  | VL_Cat | -2.30187(1.566288) | (-5.41178, 0.80803) | 0.145 | 0.74 |
|  | HIV_Stage | 0.574388(0.576003) | (-0.56928, 1.718056) | 0.321 | 0.84 |
|  | ARTMonths_cat | -0.23152(0.45354) | (-1.13203, 0.668995) | 0.611 | 0.956 |
|  |  |  |  |  |  |
| **IL-1β** | Underweight | 2.160228(6.745493) | (-11.2331, 15.55356) | 0.749 | 0.963 |
|  | Overweight | 2.342634(4.393216) | (-6.3802, 11.06547) | 0.595 | 0.95 |
|  | Obese | 6.263493(6.196674) | (-6.04015, 18.56714) | 0.315 | 0.84 |
|  | SIS | 0.726289(5.475531) | (-10.1455, 11.59808) | 0.895 | 0.983 |
|  | MIS | 1.891757(3.115236) | (-4.29362, 8.077131) | 0.545 | 0.95 |
|  | Underweight X MIS | -0.2203(9.144161) | (-18.3763, 17.93564) | 0.981 | 0.983 |
|  | Overweight X SIS | 0((empty)) | (, ) |  |  |
|  | Overweight X MIS | -7.80969(7.680374) | (-23.0593, 7.439873) | 0.312 | 0.84 |
|  | Obese X SIS | -1.85339(14.58019) | (-30.8027, 27.09593) | 0.899 | 0.983 |
|  | Obese X MIS | -8.19969(9.537282) | (-27.1362, 10.73681) | 0.392 | 0.862 |
|  | Sex | -2.91868(2.617984) | (-8.11675, 2.279391) | 0.268 | 0.793 |
|  | CC | -2.56543(2.547426) | (-7.6234, 2.492547) | 0.316 | 0.84 |
|  | VL_Cat | -3.51441(3.742117) | (-10.9445, 3.915651) | 0.35 | 0.853 |
|  | HIV_Stage | -0.46092(1.376165) | (-3.19333, 2.271488) | 0.738 | 0.963 |
|  | ARTMonths_cat | 0.603797(1.08358) | (-1.54768, 2.75527) | 0.579 | 0.95 |
|  |  |  |  |  |  |
| **IFN-γ** | Underweight | -0.49573(11.71113) | (-23.7485, 22.757) | 0.966 | 0.983 |
|  | Overweight | 1.258447(7.627247) | (-13.8856, 16.40252) | 0.869 | 0.983 |
|  | Obese | 0.660628(10.75831) | (-20.7002, 22.0215) | 0.951 | 0.983 |
|  | SIS | 10.84589(9.506299) | (-8.02909, 29.72087) | 0.257 | 0.793 |
|  | MIS | 0.52341(5.408492) | (-10.2153, 11.2621) | 0.923 | 0.983 |
|  | Underweight X MIS | 7.973712(15.87556) | (-23.5476, 39.49501) | 0.617 | 0.956 |
|  | Overweight X SIS | 0((empty)) | (, ) |  |  |
|  | Overweight X MIS | -2.13622(13.33422) | (-28.6116, 24.33919) | 0.873 | 0.983 |
|  | Obese X SIS | 8.771262(25.31329) | (-41.4889, 59.03139) | 0.73 | 0.963 |
|  | Obese X MIS | 18.23682(16.55808) | (-14.6396, 51.11327) | 0.274 | 0.793 |
|  | Age | -0.11464(0.203226) | (-0.51815, 0.288872) | 0.574 | 0.95 |
|  | Sex | -0.09238(4.545192) | (-9.11697, 8.932206) | 0.984 | 0.984 |
|  | CC | 3.970792(4.422693) | (-4.81057, 12.75215) | 0.372 | 0.853 |
|  | VL_Cat | -7.79436(6.496847) | (-20.694, 5.10528) | 0.233 | 0.776 |
|  | HIV_Stage | 2.917712(2.389218) | (-1.82614, 7.66156) | 0.225 | 0.776 |
|  | ARTMonths_cat | -1.03376(1.881249) | (-4.76903, 2.7015) | 0.584 | 0.95 |

***β (SE) represents the regression coefficient with standard error. CI = confidence interval; BH = Benjamini-Hochberg adjusted p-value for multiple comparisons. Bold p-values indicate statistical significance at p < 0.05.***

**Stratified Analysis:**

**Table 2: Stratified Analysis of Cytokine Levels by BMI Categories (Underweight, Normal Weight, Overweight, Obese) and CD4+ Count Categories in People Living with HIV**

| **BMI category: Underweight** | |  |  |  |  |
| --- | --- | --- | --- | --- | --- |
| **Cytokine** | **variable** | **β (SE)** | **95% CI** | **p-value** | **p-value (BH)** |
| **TNF-a** | CD4_Cat | 0.160331(5.509723) | (-17.3741, 17.69473) | 0.979 | 0.985 |
|  | AgeGroup | -1.33365(2.927696) | (-10.6509, 7.983587) | 0.68 | 0.96 |
|  | Sex | -0.0966(4.87339) | (-15.6059, 15.4127) | 0.985 | 0.985 |
|  | CC | 1.756769(5.419862) | (-15.4917, 19.00519) | 0.767 | 0.985 |
|  | HIV_Stage | 2.658911(1.866047) | (-3.27968, 8.597506) | 0.249 | 0.935 |
|  |  |  |  |  |  |
| **IL-1β** | CD4_Cat | -6.14776(12.54361) | (-46.0671, 33.7716) | 0.658 | 0.954 |
|  | AgeGroup | 2.620937(6.665284) | (-18.591, 23.83285) | 0.72 | 0.963 |
|  | Sex | 11.90675(11.09491) | (-23.4022, 47.21572) | 0.362 | 0.89 |
|  | CC | 2.143744(12.33903) | (-37.1246, 41.41204) | 0.873 | 0.985 |
|  | HIV_Stage | 5.25757(4.248302) | (-8.26242, 18.77756) | 0.304 | 0.873 |
|  |  |  |  |  |  |
| **IFN-γ** | CD4_Cat | -12.7371(23.99633) | (-89.1042, 63.62992) | 0.632 | 0.944 |
|  | AgeGroup | 5.050771(12.75091) | (-35.5283, 45.62985) | 0.719 | 0.962 |
|  | Sex | 19.08181(21.22493) | (-48.4654, 86.62901) | 0.435 | 0.923 |
|  | CC | 16.71946(23.60497) | (-58.4021, 91.841) | 0.53 | 0.944 |
|  | HIV_Stage | 9.62681(8.12714) | (-16.2374, 35.491) | 0.321 | 0.84 |
|  |  |  |  |  |  |
| **BMI category: Normal Weight** | |  |  |  |  |
| **Cytokine** | **variable** | **β (SE)** | **95% CI** | **p-value** | p-value (BH) |
| **TNF-a** | CD4_Cat | -0.99889(1.103649) | (-3.20061, 1.202825) | 0.369 | 0.89 |
|  | AgeGroup | -0.18603(0.967424) | (-2.11599, 1.743931) | 0.848 | 0.985 |
|  | Sex | 0.603413(1.403036) | (-2.19557, 3.402393) | 0.668 | 0.96 |
|  | CC | -0.23344(1.352907) | (-2.93241, 2.46554) | 0.864 | 0.985 |
|  | HIV_Stage | 0.42179(0.738485) | (-1.05145, 1.895028) | 0.57 | 0.935 |
|  |  |  |  |  |  |
| **IL-1β** | CD4_Cat | 0.084562(2.471714) | (-4.84637, 5.015496) | 0.973 | 0.985 |
|  | AgeGroup | -0.53611(2.166627) | (-4.85841, 3.786194) | 0.805 | 0.973 |
|  | Sex | -2.17664(3.142215) | (-8.44518, 4.091911) | 0.491 | 0.934 |
|  | CC | -4.67642(3.029947) | (-10.721, 1.368163) | 0.127 | 0.586 |
|  | HIV_Stage | -0.63331(1.653899) | (-3.93275, 2.666127) | 0.703 | 0.96 |
|  |  |  |  |  |  |
| **IFN-γ** | CD4_Cat | -2.3185(4.330018) | (-10.9567, 6.31965) | 0.594 | 0.935 |
|  | AgeGroup | -1.5698(3.795558) | (-9.14174, 6.002129) | 0.68 | 0.96 |
|  | Sex | -1.89944(5.504621) | (-12.8809, 9.081984) | 0.731 | 0.963 |
|  | CC | -1.25052(5.307947) | (-11.8396, 9.338546) | 0.814 | 0.974 |
|  | HIV_Stage | 3.045294(2.897348) | (-2.73476, 8.825345) | 0.297 | 0.855 |
|  |  |  |  |  |  |
| **BMI category: Overweight** | |  |  |  |  |
| **Cytokine** | **variable** | **β (SE)** | **95% CI** | **p-value** | p-value (BH) |
| **TNF-a** | CD4_Cat | 2.926616(2.631065) | (-2.86432, 8.717552) | 0.29 | 0.806 |
|  | AgeGroup | -3.56538(1.583101) | (-7.04977, -0.081) | 0.046 | 0.391 |
|  | Sex | -0.42807(1.747985) | (-4.27536, 3.419224) | 0.811 | 0.973 |
|  | CC | -0.97305(1.894511) | (-5.14284, 3.196737) | 0.618 | 0.944 |
|  | HIV_Stage | -3.09978(1.179521) | (-5.69589, -0.50367) | 0.023 | 0.351 |
|  |  |  |  |  |  |
| **IL-1β** | CD4_Cat | 12.45293(8.814532) | (-6.94772, 31.85359) | 0.185 | 0.873 |
|  | AgeGroup | -3.38546(5.303668) | (-15.0588, 8.287841) | 0.536 | 0.923 |
|  | Sex | -3.65964(5.856059) | (-16.5487, 9.22946) | 0.545 | 0.924 |
|  | CC | 0.599845(6.346945) | (-13.3697, 14.56938) | 0.926 | 0.985 |
|  | HIV_Stage | -6.47375(3.951603) | (-15.1712, 2.22367) | 0.13 | 0.741 |
|  |  |  |  |  |  |
| **IFN-γ** | CD4_Cat | 12.41451(13.62907) | (-17.5829, 42.41189) | 0.382 | 0.873 |
|  | AgeGroup | -17.6767(8.200558) | (-35.726, 0.372567) | 0.054 | 0.391 |
|  | Sex | -1.9969(9.054666) | (-21.9261, 17.93228) | 0.829 | 0.923 |
|  | CC | 4.057137(9.813676) | (-17.5426, 25.65689) | 0.687 | 0.89 |
|  | HIV_Stage | -13.2955(6.109987) | (-26.7435, 0.152481) | 0.052 | 0.39 |
|  |  |  |  |  |  |
| **BMI category: Obese** | |  |  |  |  |
| **Cytokine** | **variable** | **β (SE)** | **95% CI** | **p-value** | p-value (BH) |
| **TNF-a** | CD4_Cat | -3.10165(1.437096) | (-7.67513, 1.471835) | 0.12 | 0.18 |
|  | AgeGroup | -1.25662(1.218357) | (-5.13398, 2.620736) | 0.378 | 0.529 |
|  | Sex | 2.284341(1.919399) | (-3.82405, 8.392726) | 0.32 | 0.48 |
|  | CC | 0.105934(1.667498) | (-5.20079, 5.412657) | 0.953 | 0.953 |
|  | HIV_Stage | 3.250596(1.193831) | (-0.54871, 7.049901) | 0.072 | 0.18 |
|  |  |  |  |  |  |
| **IL-1β** | CD4_Cat | 5.731275(1.65065) | (0.47817, 10.98438) | 0.04 | 0.12 |
|  | AgeGroup | 0.172085(1.399406) | (-4.28145, 4.62562) | 0.91 | 0.953 |
|  | Sex | -13.9228(2.204625) | (-20.9389, -6.90667) | 0.008 | 0.04 |
|  | CC | 0.971838(1.91529) | (-5.12347, 7.067146) | 0.647 | 0.809 |
|  | HIV_Stage | 8.294859(1.371236) | (3.930973, 12.65874) | 0.009 | 0.04 |
|  |  |  |  |  |  |
| **IFN-γ** | CD4_Cat | -17.9632(13.3782) | (-60.5386, 24.61218) | 0.272 | 0.48 |
|  | AgeGroup | -6.85114(11.34192) | (-42.9462, 29.24391) | 0.588 | 0.84 |
|  | Sex | 5.136374(17.86805) | (-51.7278, 62.0005) | 0.792 | 0.953 |
|  | CC | 17.20242(15.52306) | (-32.1989, 66.60371) | 0.349 | 0.529 |
|  | HIV_Stage | 15.49161(11.1136) | (-19.8768, 50.86006) | 0.258 | 0.48 |

**β (SE) represents the regression coefficient with standard error. CI = confidence interval; BH = Benjamini-Hochberg adjusted p-value. Results are stratified by BMI categories (underweight, normal weight, overweight, obese) and CD4+ count categories. Bold p-values indicate statistical significance at p < 0.05**

**Table 3: Multivariate Regression and Interaction Analysis of Viral Load and Sex on Cytokine Levels**

| **Cytokine** | **variable** | **β (SE)** | **95% CI** | **p-value** | p-value (BH) |
| --- | --- | --- | --- | --- | --- |
| **TNF-a** | Underweight | -1.33177(1.820212) | (-4.94438, 2.280851) | 0.466 | 0.888 |
|  | Overweight | -0.66697(1.423574) | (-3.49237, 2.15843) | 0.64 | 0.888 |
|  | Obese | -1.73815(1.864862) | (-5.43938, 1.963088) | 0.354 | 0.888 |
|  | SIS | 3.155842(2.121168) | (-1.05409, 7.365773) | 0.14 | 0.780 |
|  | MIS | 0.8155(1.112056) | (-1.39162, 3.022623) | 0.465 | 0.888 |
|  | Age Group | -0.46645(0.750558) | (-1.9561, 1.023201) | 0.536 | 0.888 |
|  | Sex | 0.170232(1.13937) | (-2.0911, 2.431566) | 0.882 | 0.952 |
|  | VL_Cat (Detectable) | -3.08585(1.927521) | (-6.91145, 0.739743) | 0.113 | 0.753 |
|  | Female X Detectable VL | 2.010982(3.1488) | (-4.23852, 8.26048) | 0.525 | 0.888 |
|  | CC+ | 0.358046(1.030574) | (-1.68736, 2.403449) | 0.729 | 0.888 |
|  | HIV_Stage | 0.575166(0.538401) | (-0.49341, 1.643743) | 0.288 | 0.888 |
|  | ARTMonths_cat | -0.26896(0.45016) | (-1.1624, 0.624481) | 0.552 | 0.888 |
|  |  |  |  |  |  |
| **IL-1β** | Underweight | 0.944504(4.323084) | (-7.63562, 9.52463) | 0.828 | 0.952 |
|  | Overweight | -0.81711(3.381052) | (-7.52756, 5.893348) | 0.81 | 0.952 |
|  | Obese | 3.285381(4.42913) | (-5.50522, 12.07598) | 0.46 | 0.888 |
|  | SIS | 0.648923(5.037868) | (-9.34985, 10.6477) | 0.898 | 0.952 |
|  | MIS | -0.16504(2.641182) | (-5.40706, 5.076973) | 0.95 | 0.952 |
|  | Age Group | -1.26958(1.782608) | (-4.80756, 2.268407) | 0.478 | 0.888 |
|  | Sex | -3.88439(2.706054) | (-9.25516, 1.486381) | 0.154 | 0.780 |
|  | VL_Cat (Detectable) | -7.5846(4.577948) | (-16.6706, 1.501363) | 0.101 | 0.753 |
|  | Female X Detectable VL | 10.96284(7.478541) | (-3.88, 25.80567) | 0.146 | 0.780 |
|  | CC+ | -2.70436(2.447659) | (-7.56229, 2.153564) | 0.272 | 0.888 |
|  | HIV_Stage | -0.07449(1.278727) | (-2.61241, 2.463428) | 0.954 | 0.952 |
|  | ARTMonths_cat | 0.803832(1.06915) | (-1.31814, 2.925798) | 0.454 | 0.888 |
|  |  |  |  |  |  |
| **IFN-γ** | Underweight | 2.880875(7.540281) | (-12.0845, 17.84625) | 0.703 | 0.888 |
|  | Overweight | 0.915856(5.897198) | (-10.7885, 12.62016) | 0.877 | 0.952 |
|  | Obese | 8.760094(7.725244) | (-6.57238, 24.09257) | 0.26 | 0.780 |
|  | SIS | 12.92451(8.786999) | (-4.51526, 30.36427) | 0.145 | 0.780 |
|  | MIS | 1.696775(4.606724) | (-7.4463, 10.83985) | 0.713 | 0.888 |
|  | Age Group | -2.2359(3.109208) | (-8.40681, 3.935021) | 0.474 | 0.888 |
|  | Sex | -2.64601(4.719874) | (-12.0137, 6.721637) | 0.576 | 0.888 |
|  | VL_Cat (Detectable) | -13.2653(7.984811) | (-29.1129, 2.582339) | 0.1 | 0.753 |
|  | Female X Detectable VL | 14.80832(13.044) | (-11.0804, 40.69704) | 0.259 | 0.780 |
|  | CC+ | 4.250591(4.269182) | (-4.22255, 12.72374) | 0.322 | 0.888 |
|  | HIV_Stage | 2.894077(2.230343) | (-1.53254, 7.320691) | 0.198 | 0.888 |
|  | ARTMonths_cat | -0.64326(1.864801) | (-4.34438, 3.05785) | 0.731 | 0.888 |

**β (SE) represents the regression coefficient with standard error. CI = confidence interval; BH = Benjamini-Hochberg adjusted p-value. Interaction terms evaluate the modifying effect of viral load on the relationship between sex and cytokine levels. Bold p-values indicate statistical significance at p < 0.05**
